# CCR9 axis inhibition enhances hepatic migration of plasmacytoid DCs and protects against liver injury

**DOI:** 10.1172/jci.insight.159910

**Published:** 2022-09-08

**Authors:** Yuzo Koda, Nobuhiro Nakamoto, Po-Sung Chu, Toshiaki Teratani, Akihisa Ueno, Takeru Amiya, Nobuhito Taniki, Sayako Chiba, Kentaro Miyamoto, Michiie Sakamoto, Takanori Kanai

**Affiliations:** 1 Division of Gastroenterology and Hepatology, Department of Internal Medicine, Keio University School of Medicine, Tokyo, Japan.; 2 Mitsubishi Tanabe Pharma Corporation, Kanagawa, Japan.; 3 Department of Pathology, Keio University School of Medicine, Tokyo, Japan.; 4 Japan Agency for Medical Research and Development, Tokyo, Japan.

**Keywords:** Hepatology, Immunology, Chemokines, Dendritic cells, Hepatitis

## Abstract

Plasmacytoid dendritic cells (pDCs) perform dual proinflammatory and immunosuppressive roles. We recently reported the potential of pDC therapy for treatment of intractable acute liver failure. However, establishment of efficient methods to deliver pDCs to the liver is essential for future clinical therapeutic applications. The present study demonstrates a higher abundance of liver and peripheral blood pDCs in mice lacking C-C motif chemokine receptor 9 (CCR9), a pDC gut-homing receptor, than in WT mice. Adoptive transfer of *Ccr9^–/–^* pDCs resulted in a higher efficiency of migration to the liver than WT pDCs did, while WT pDCs migrated efficiently to the original target organ, the small intestine. Further, *Ccr9^–/–^* pDCs consistently migrated efficiently to livers with concanavalin A–induced inflammation, and exerted a more effective immunosuppressive effect, resulting in better protection against acute liver inflammation than that demonstrated by WT pDCs. These findings highlight the therapeutic potential of the manipulation of the CCR9 axis as an approach to improve migration of immunosuppressive pDCs to the liver in order to exploit their beneficial effects in acute liver disease.

## Graphical abstract

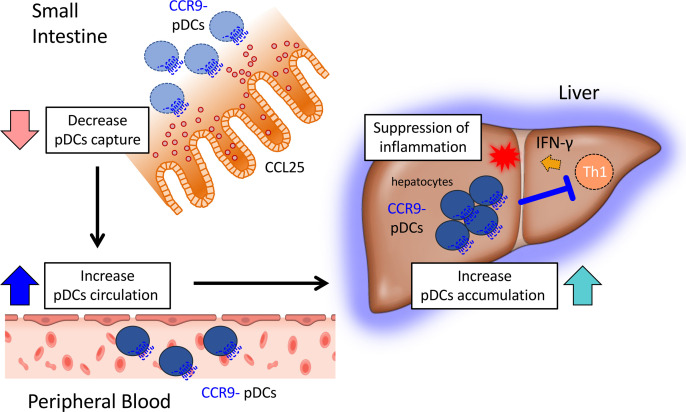

## Introduction

The liver is well recognized as an immunologically tolerant organ that limits excessive response to food-derived antigens and bacterial products in the portal vein circulation (1, 2). Both innate immunity and adaptive immunity play key roles in local and systemic responses against pathogens, while simultaneously promoting self-tolerance (3). Various immune cells are known to participate in organ-specific hepatic immune tolerance in both healthy and diseased conditions (4–6).

Plasmacytoid dendritic cells (pDCs) were first identified as a cellular subset with antiviral properties in 1999 (7, 8). Mature pDCs produce type I IFN via TLR7 and TLR9 signaling, thus contributing to viral elimination and local inflammation (9). The immunosuppressive activity of pDCs is known in several diseases, including graft-versus-host disease, allergic disease, atherosclerosis, enteritis, and colitis (10–13). Hepatic pDCs are abundant and exert stronger immunosuppressive effects via IL-10, IL-27, and PD-L1 signaling than do pDCs at other sites (14–16). Recently, we demonstrated the protective role of pDCs in concert with Tregs and IL-35 against immune-mediated acute liver injury in humans and mice (17), which positions pDC-based therapy as a potential novel therapeutic approach for intractable acute liver injuries including acute liver failure (ALF). However, the preparation of pDCs in numbers sufficient for clinical use is challenging, as they represent less than 1% of peripheral blood mononuclear cells, which is further reduced in ALF. This necessitates the standardization of appropriate methodologies to improve the growth and differentiation of pDCs, and their efficient delivery to the liver.

The migration and accumulation of immune cells in the liver are regulated by organ-specific chemokines and receptors including CXCR3 and CXCR6 under physiological conditions (18). Under inflammatory conditions, both inflammatory and suppressive cells are recruited by chemokine axes such as the CCR2/CCL2 and CXCR4/CXCL12 axes (19, 20). Recent reports have shown that the intestinal microbiome also controls the hepatic immune cell population (21, 22). Several strategies exist for efficiently delivering cells from nontarget to target organs. For instance, inhibition of the CXCR4/CXCL12 axis via administration of plerixafor, a CXCR4 antagonist, results in the release of hematopoietic stem cells into the bloodstream (23). This well-known technique is widely applied in clinical settings for stem cell transplantation (24). Despite the proposal of different strategies to manipulate chemokine axes (25, 26), no optimized method has been demonstrated thus far to improve the efficacy of immune cell migration to the liver.

C-C motif chemokine receptor 9 (CCR9), a gut-homing chemokine receptor, is expressed at high levels in pDCs. Wendland et al. showed that CCR9 deficiency causes pDC ablation in the small intestine (27). Because pDCs are most abundant in the small intestine, we hypothesized that inhibition of pDC migration to this organ may result in increased blood circulation, and subsequent migration of these cells to the liver, which is also a target organ abundant in pDCs. In the present study, we demonstrate that *Ccr9^–/–^* mice exhibit increased peripheral blood (PB) circulation and liver-specific accumulation of pDCs. Further, we show that the adoptive transfer of *Ccr9^–/–^* pDCs resulted in better protection against concanavalin A–induced (ConA-induced) acute liver injury than that achieved by the transfer of WT pDCs, suggesting the possibility of CCR9-independent pDC migration to the inflamed liver.

## Results

### CCR9 deficiency results in enhanced accumulation of pDCs in normal and inflamed livers.

**Figure 1:**
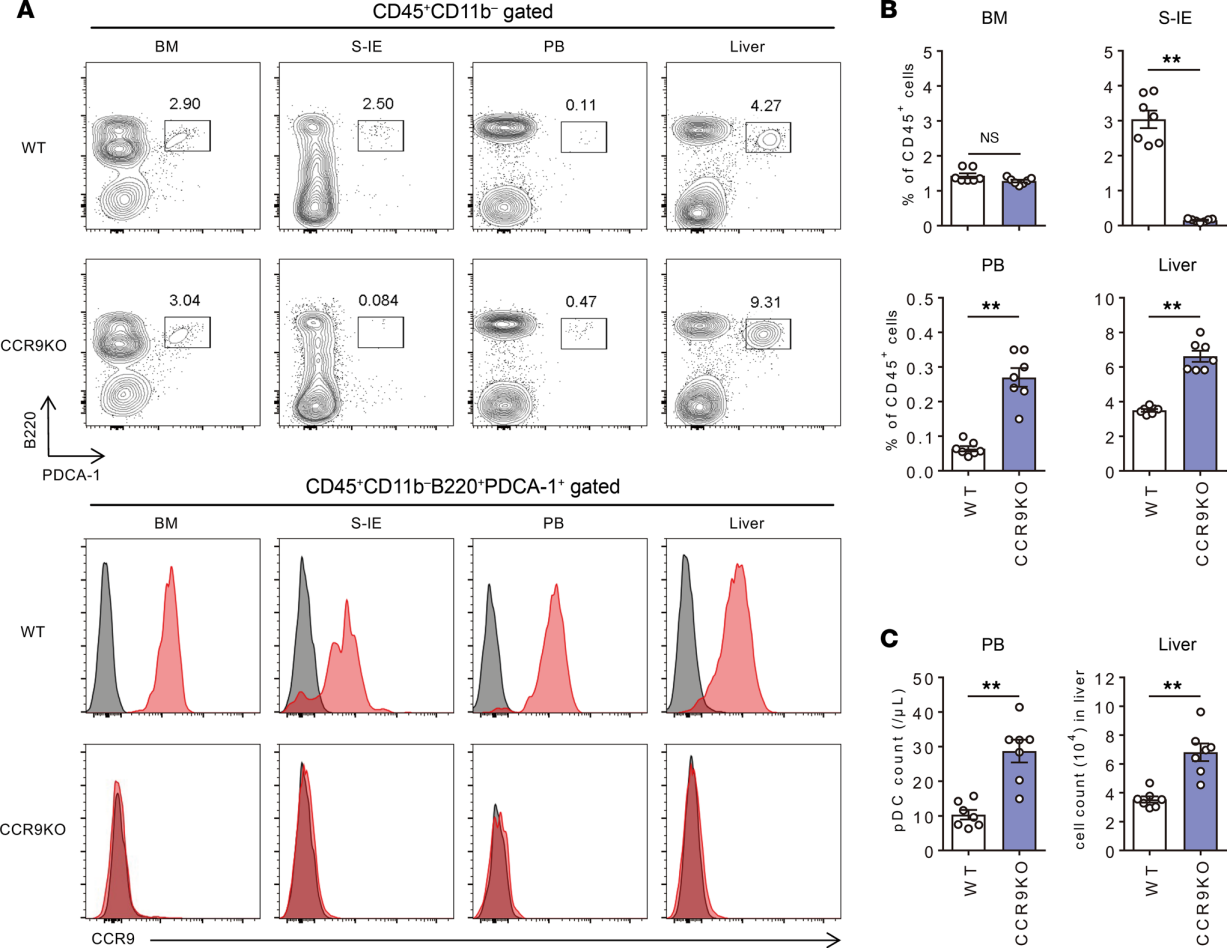
CCR9 deficiency induces hepatic pDC accumulation under steady-state conditions. (**A**) Top: Representative B220 and PDCA-1 staining of CD45^+^CD11b^–^-gated BM, S-IE, and PB mononuclear cells (MNCs) in male WT or *Ccr9^–/–^* mice with C57BL/6 background. Bottom: Representative histograms showing CCR9 expression in hepatic CD45^+^CD11b^–^B220^+^PDCA-1^+^ pDCs. (**B**) Mean percentages of pDCs in the CD45^+^ MNC population from BM, S-IE, PB, and livers of WT or *Ccr9^–/–^* mice. Data represent the mean ± SEM (*n* = 7 per group). (**C**) Absolute numbers of pDCs in PB and liver. Data represent the mean ± SEM (*n* = 7 per group). **P < 0.01, Student’s *t* test. Data are combinations of 2 independent experiments from over 5 independent experiments (**B** and **C**).

**Figure 2:**
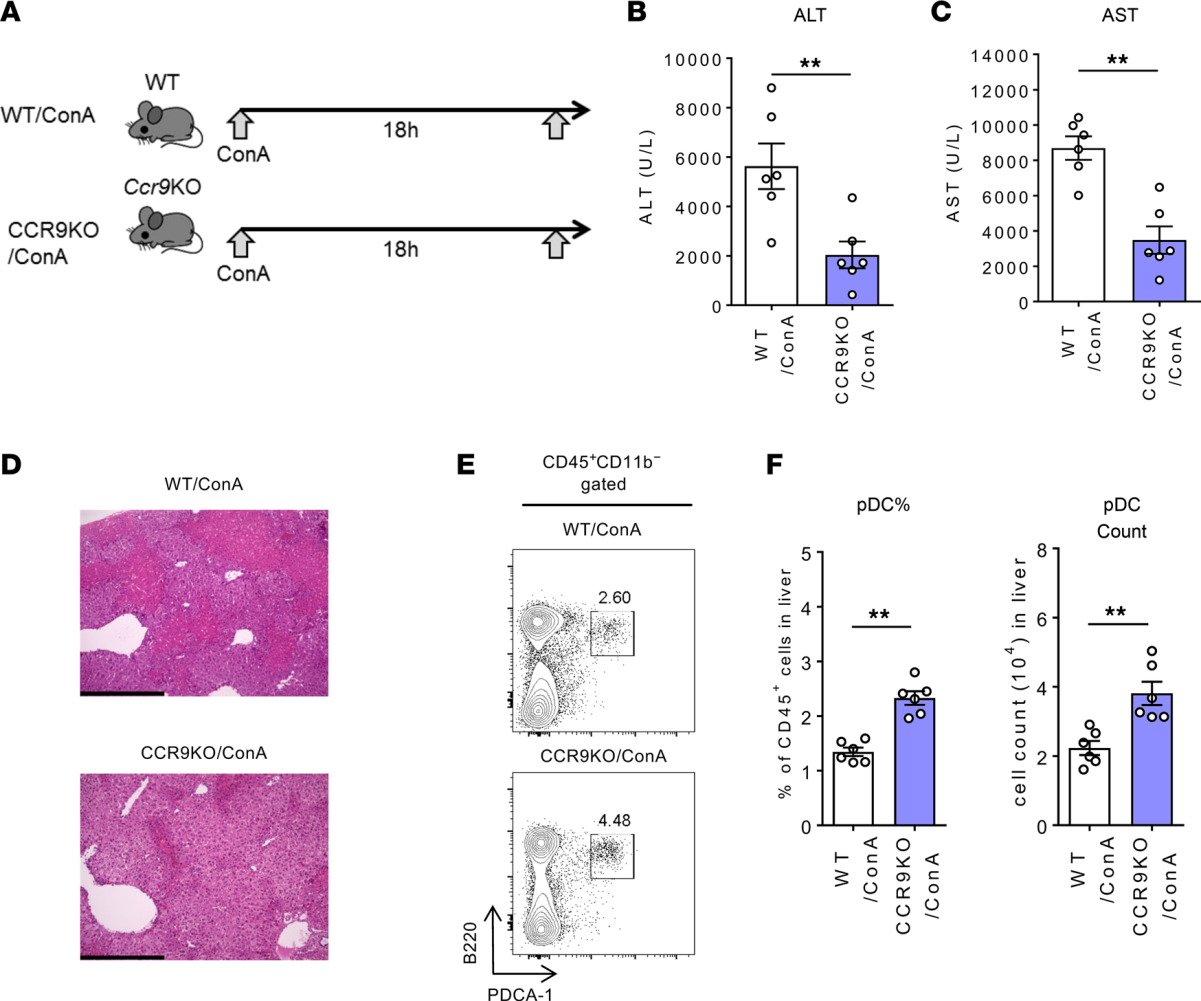
CCR9 deficiency induces hepatic pDC accumulation in ConA-induced inflammation. (**A**) Study design. WT or *Ccr9^–/–^* mice were injected i.v. with ConA (15 mg/kg) or PBS. All mice were sacrificed and analyzed 18 hours after ConA injection. (**B**) Serum alanine aminotransferase (ALT) levels. (**C**) Serum aspartate aminotransferase (AST) levels. (**D**) Representative photomicrographs of H&E-stained liver sections. Scale bars: 500 μm. (**E**) Representative B220 and PDCA-1 staining of CD45^+^CD11b^–^-gated hepatic MNCs. (**F**) Mean percentages (left) and numbers (right) of hepatic pDCs in ConA-induced hepatitis. Data represent the mean ± SEM (*n* = 6 per group). **P < 0.01, Student’s *t* test. Data are combinations of 2 independent experiments (**B**, **C**, and **F**).

Earlier reports have demonstrated a significant decrease in the number of pDCs in the small intestine of *Ccr9^–/–^* mice (27). However, the pDC distribution in other organs of *Ccr9^–/–^* mice has not been previously described. We therefore made a simultaneous comparison of the distribution of pDCs and CCR9 expression in various tissues of WT and *Ccr9^–/–^* mice. For this purpose, pDCs were defined as CD45^+^CD11b^–^B220^+^PDCA-1^+^ cells. The gating strategy for the FACS analysis used in this study is shown in Supplemental Figure 1 (supplemental material available online with this article; https://doi.org/10.1172/jci.insight.159910DS1). The findings revealed high levels of CCR9 expression in pDCs of the BM, small intestinal epithelium (S-IE), PB, and liver (Figure 1A). Notably, the proportion of pDCs in the small intestine of *Ccr9^–/–^* mice was decreased, whereas that of pDCs in the liver and PB was significantly increased (Figure 1, B and C). Consistent with a previous report (28), serological and histological analysis to determine the effect of CCR9 deficiency on pDC accumulation in the liver of mice with ConA-induced acute liver injury revealed a significant reduction in liver inflammation after ConA administration in *Ccr9^–/–^* mice (Figure 2, A–D). Additionally, both the proportion and total number of hepatic pDCs were consistently increased in *Ccr9^–/–^* mice (Figure 2, E and F). The hepatic accumulation of pDCs in inflammatory conditions was also confirmed in a second mouse model with acute liver injury induced by carbon tetrachloride (CCl_4_) (Supplemental Figure 2). Furthermore, CCL25 inhibition resulted in the enrichment of hepatic pDCs and in partial improvement of hepatobiliary injuries in MDR2^–/–^ mice (Supplemental Figure 3). These results suggest that CCR9/CCL25 deficiency enhances pDC accumulation in normal and inflamed livers, and potentially results in less severe liver inflammation.

### CCR9 deficiency does not alter the phenotype of hepatic pDCs.

**Figure 3:**
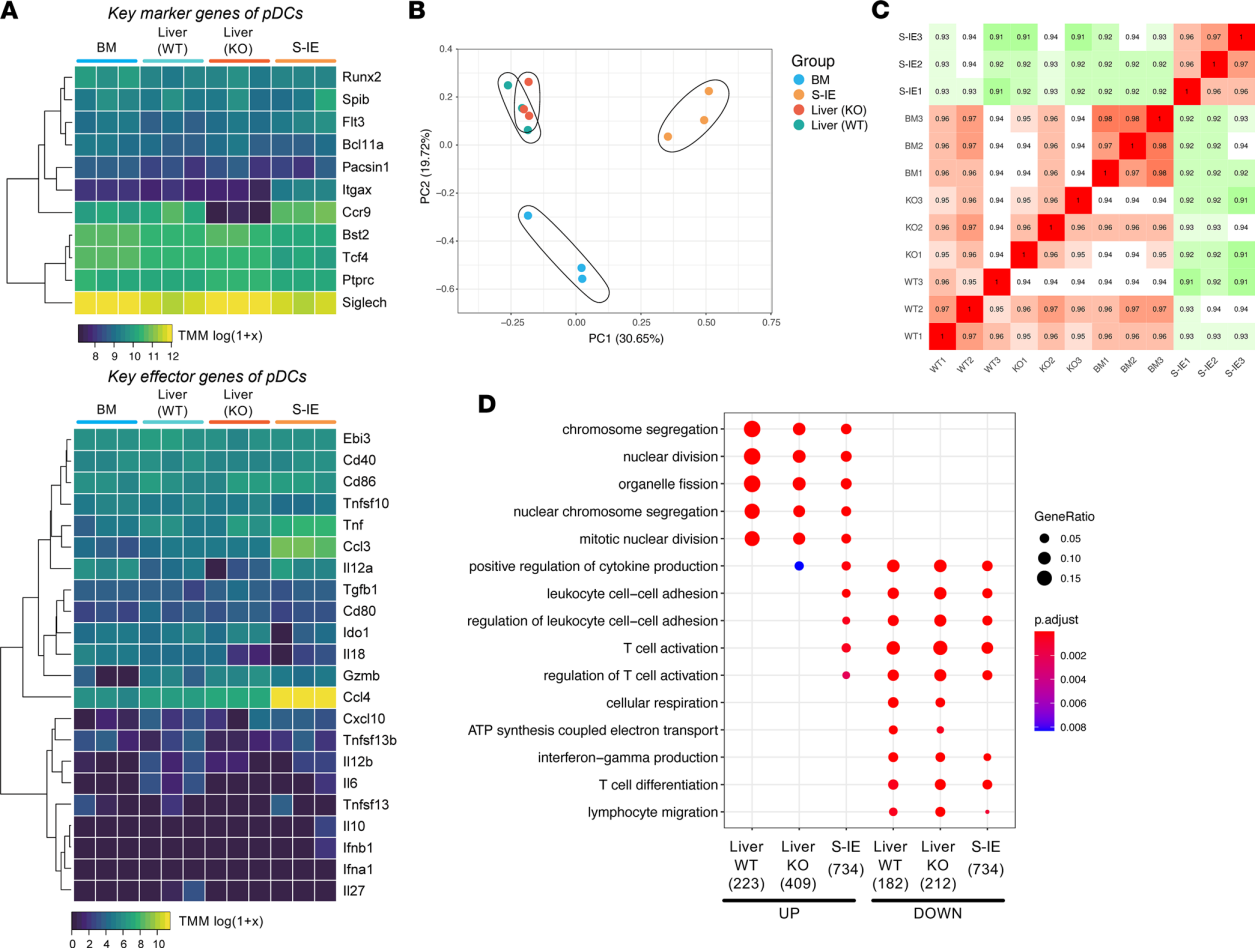
RNA-Seq analysis of hepatic pDCs derived from WT or *Ccr9^–/–^* mice. Gene expression profiling of the liver (WT or *Ccr9^–/–^* mice), BM (WT mice), and SI-E (WT mice) pDCs was performed using RNA-Seq analysis (*n* = 3). CD45^+^CD11b^–^B220^+^PDCA-1^+^Siglec-H^+^ cells were isolated from each organ. (**A**) Heatmap of pDC markers (top) and effector genes (bottom) of pDCs from each tissue. (**B**) Principal component analysis (PCA) plot of the top 2,000 genes. (**C**) Pearson’s correlation matrix using the top 75% of genes. (**D**) GO enrichment analysis of hepatic pDCs (WT or *Ccr9*^–/–^) and IE pDCs (WT) in comparison with BM pDCs (WT). Columns show enriched GO terms from upregulated genes (left 3 columns) and downregulated genes (right 3 columns).

The organ-specific gene expression profiles of pDCs derived from BM and S-IE of WT mice and the effect of CCR9 deficiency on the phenotype of hepatic pDCs from WT or *Ccr9^–/–^* mice were examined by RNA-Seq analysis. As shown in Figure 3, A–C, the gene expression profiles clearly differed between the tissues examined. In particular, the expression of inflammatory genes such as *Tnf*, *Ccl3*, and *Ccl4* was significantly upregulated in S-IE pDCs. Gene Ontology (GO) enrichment analysis further confirmed that S-IE pDCs showed higher levels of activation and inflammation as compared with that seen in BM and liver pDCs (Figure 3D). However, no significant change in the gene expression profiles of hepatic pDCs consequent to CCR9 deficiency was observed (Figure 3, A–D). These findings suggest that although CCR9 regulates pDC dynamics, its deficiency does not affect the nature of pDCs.

### Ccr9^–/–^ pDCs efficiently migrate to the liver.

**Figure 4:**
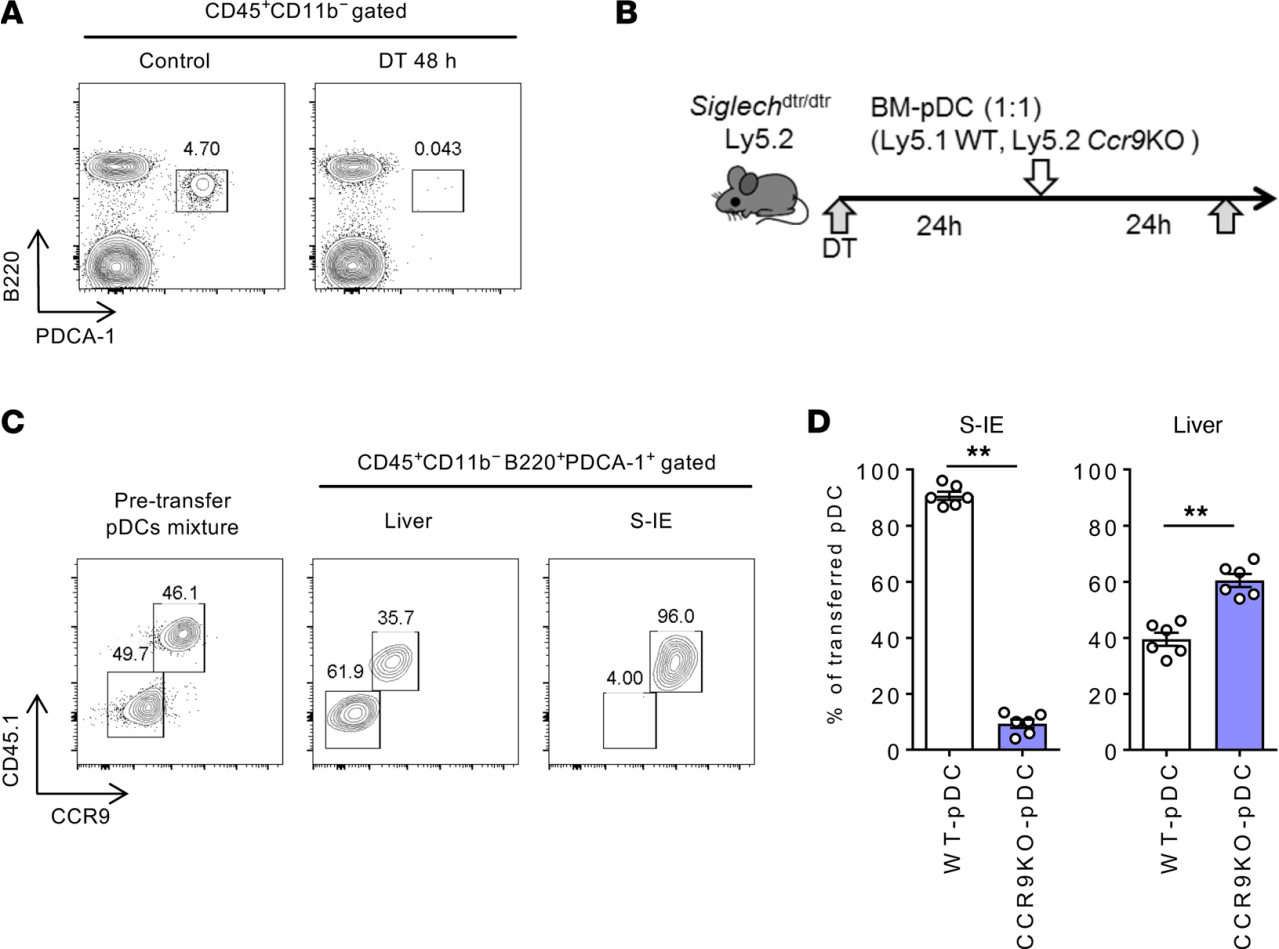
Adoptive transfer of *Ccr9^–/–^* pDCs results in efficient migration of pDCs to the liver under steady-state conditions. (**A**) WT or *Siglech^dtr/dtr^* mice were treated with diphtheria toxin (DT; 1 μg/mouse) 48 hours before sacrifice. Representative B220 and PDCA-1 staining of CD45^+^CD11b^–^ liver MNCs of WT (left) or *Siglech^dtr/dtr^* (right) mice. (**B**) Study design. *Siglech^dtr/dtr^* mice were treated with DT (1 μg/mouse), followed by i.v. inoculation with a cell suspension (2 × 10^6^ cells/200 μL PBS) of FLT3L-proliferated pDCs derived from WT (Ly5.1) mice and *Ccr9^–/–^* (Ly5.2) mice 24 hours later. All mice were sacrificed and analyzed 24 hours after pDC inoculation. (**C**) Representative CD45.1 and CCR9 staining of the pDC mixture prior to the treatment (left) and CD45^+^CD11b^–^-gated liver and S-IE MNCs after transplantation (middle and right). (**D**) Mean percentages of WT (Ly5.1)/*Ccr9^–/–^* (Ly5.2) pDCs in the S-IE and liver after transplantation. Data represent the mean ± SEM (*n* = 6 per group). **P < 0.01, Student’s *t* test. Data are combinations of 2 independent experiments (**D**).

The direct effect of CCR9 expression in pDCs on their migration to the liver was examined per the strategy described below. After the depletion of endogenous pDCs by administration of diphtheria toxin to sialic acid–binding Ig-like lectin H (*Siglech*)^dtr/dtr^ mice, an identical number of BM pDCs from WT (Ly5.1) and *Ccr9^–/–^* (Ly5.2) mice were mixed and intravenously transferred into pDC-ablated animals (Figure 4, A and B). The majority of pDCs that migrated to the small intestine were found to be CCR9^+^ pDCs derived from WT mice. In contrast, *Ccr9^–/–^* pDCs migrated more efficiently to the liver than did WT pDCs (Figure 4, C and D). These results suggest that the small intestinal accumulation of pDCs is regulated by the CCR9/CCL25 axis, which may be exploited for efficient hepatic pDC accumulation.

### CCR9 deficiency does not influence pDC suppressive function in vitro.

**Figure 5:**
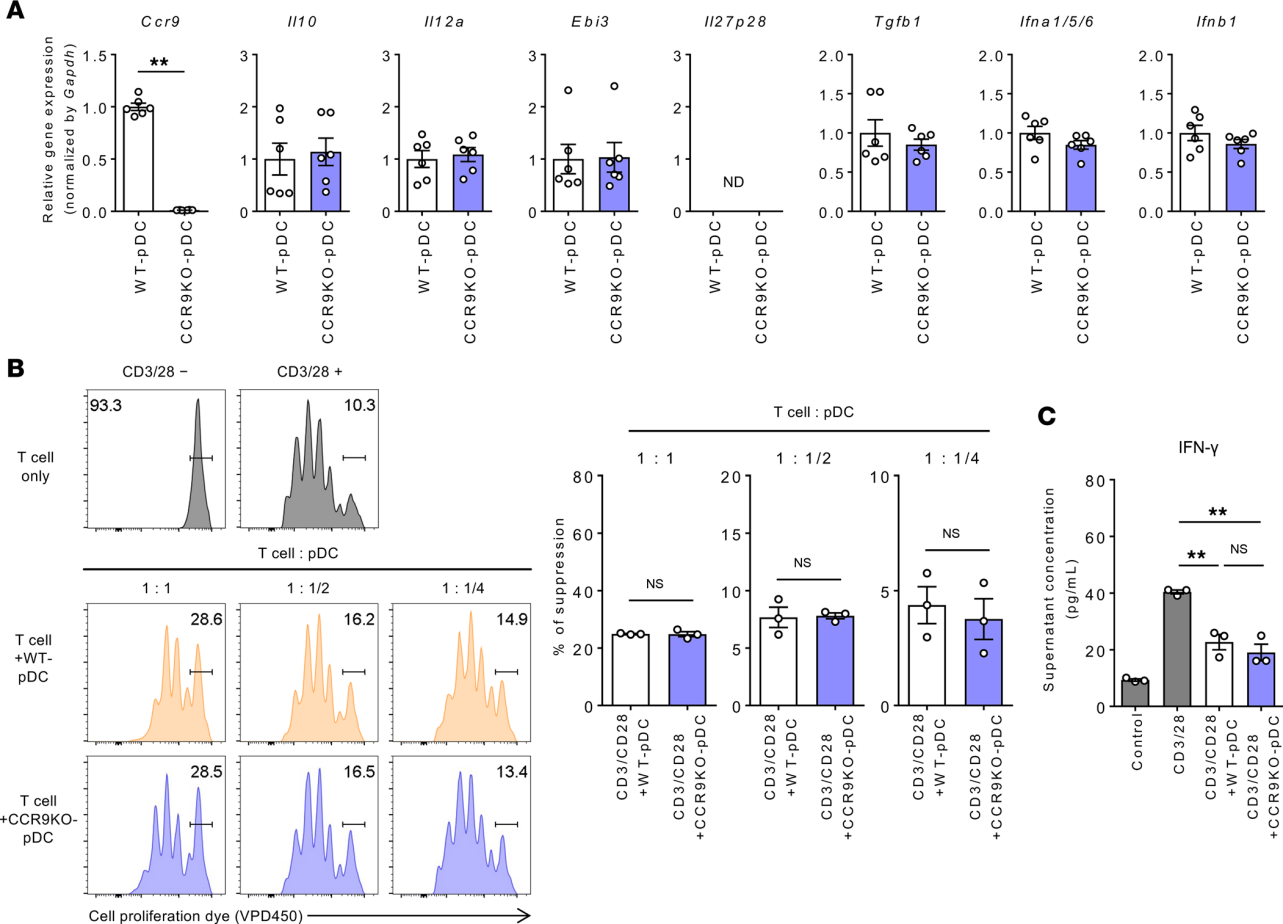
CCR9 deficiency does not influence the pDC immunosuppressive function. (**A**) Expression of various genes in FLT3L-proliferated pDCs derived from WT or *Ccr9^–/–^* mice. Data represent the mean ± SEM (*n* = 6 per group). **P < 0.01, Student’s *t* test. (**B**) FLT3L-proliferated pDCs derived from WT or *Ccr9^–/–^* mice (1 × 10^5^, 0.5 × 10^5^, or 0.25 × 10^5^ cells per well) were cocultured with VPD450-stained Teffs (1 × 10^5^ cells per well), followed by stimulation with CD3/CD28 microbeads for 4 days. Representative histograms of Teffs (left) and suppression rate of Teff proliferation by coculture with the indicated pDCs (right). Data represent the mean ± SEM (*n* = 3 per group). (**C**) IFN-γ concentration in the culture supernatant. Data represent the mean ± SEM (*n* = 3 per group). **P < 0.01, Student’s *t* test (**A** and **B**) or ANOVA with Tukey’s multiple-comparison post hoc test (**C**). Data are combinations of 2 independent experiments (**A**).

The expression of genes involved in immunosuppression in BM pDCs derived from WT and *Ccr9^–/–^* mice was compared to assess whether the immunosuppressive effect of pDCs is dependent on CCR9 expression. As shown in Figure 5A, no significant difference in the expression of representative pDC-related genes was observed, consistent with the RNA-Seq results. Furthermore, the ability of pDCs to suppress T cell proliferation and IFN-γ production was compared by coculturing of CCR9^+^ BM or CCR9^–^ BM pDCs with CD3/CD28-stimulated CD4^+^CD25^–^ effector T cells (Teffs) for 4 days. The results revealed that pDCs suppressed Teff proliferation and IFN-γ production, regardless of CCR9 expression (Figure 5, B and C). Collectively, these findings suggest that CCR9 expression in pDCs does not influence their immunosuppressive function.

### Adoptive transfer of Ccr9^–/–^ pDCs exerts enhanced protection against acute liver injury.

**Figure 6:**
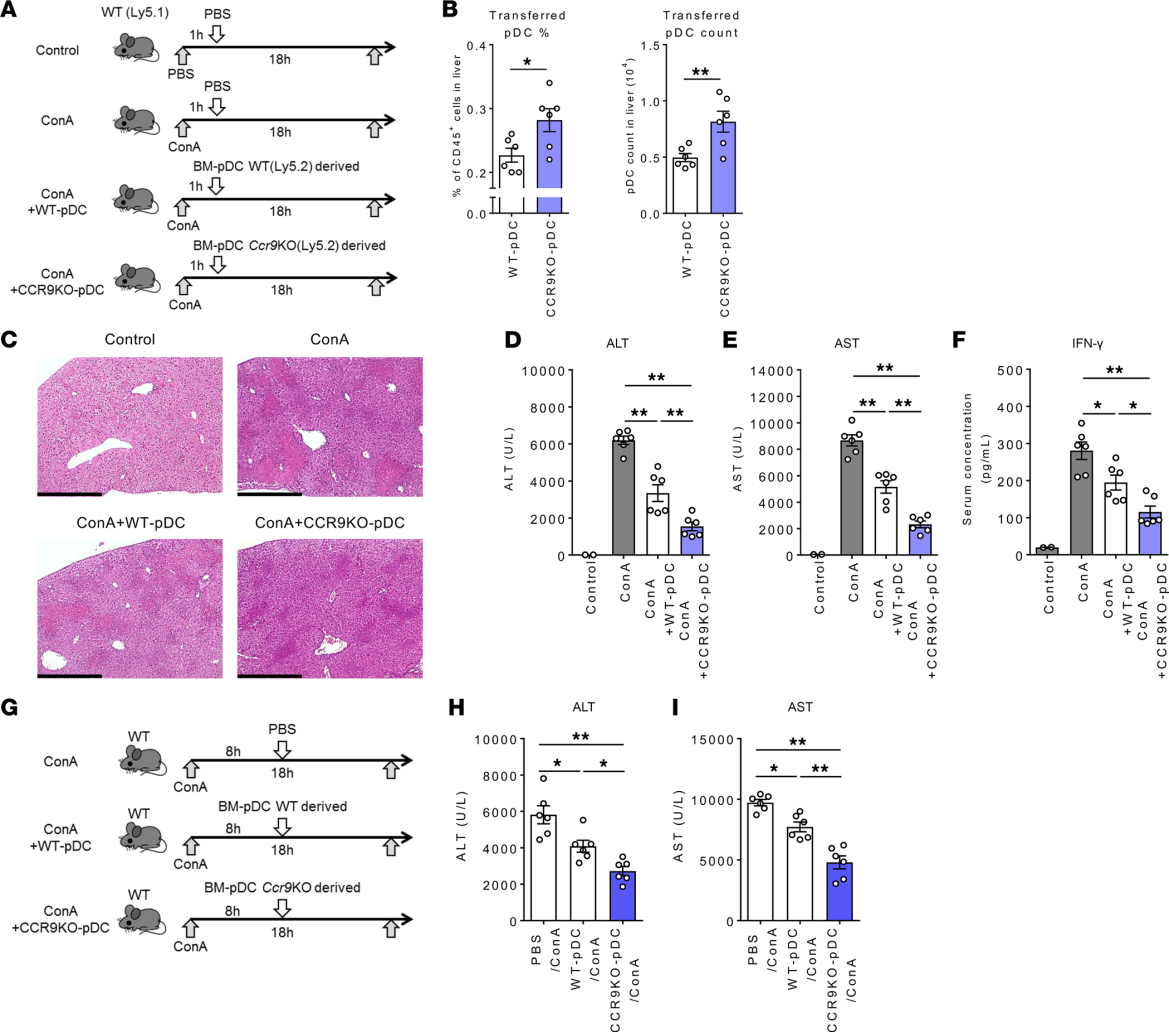
Adoptive transfer of *Ccr9^–/–^* pDCs improves protection against ConA-induced liver inflammation. (**A**) Study design. WT (Ly5.2) mice were injected i.v. with ConA (15 mg/kg) or PBS. An hour later, the mice were injected i.v. with FLT3L-proliferated BM pDCs from WT or *Ccr9^–/–^* mice (2 × 10^6^ cells/200 μL PBS) or 200 μL PBS alone. All mice were sacrificed and analyzed 18 hours after ConA injection. (**B**) Mean percentages (left) and absolute numbers (right) of transferred BM pDCs (CD45.1) in the liver during ConA-induced hepatitis. (**C**) Representative photomicrographs of H&E-stained liver sections. Scale bars: 500 μm. (**D**–**F**) Serum ALT (**D**), AST (**E**), and IFN-γ (**F**) levels. (**G**) Study design. WT mice were injected i.v. with ConA (15 mg/kg) or PBS. Eight hours later, the mice were injected i.v. with FLT3L-proliferated BM pDCs from WT or *Ccr9^–/–^* mice (2 × 10^6^ cells/200 μL PBS) or 200 μL PBS alone. All mice were sacrificed and analyzed 18 hours after ConA injection. (**H** and **I**) Serum ALT (**H**) and AST (**I**) levels. Data represent the mean ± SEM (*n* = 6 per group). *P < 0.05, **P < 0.01, Student’s *t* test (**B**) or ANOVA with Tukey’s multiple-comparison post hoc test (**D**–**F**, **H**, and **I**). Data are combinations of 2 independent experiments (**B**, **D**–**F**, **H**, and **I**).

Finally, the effect of CCR9 deficiency on the suppressive effect of pDCs in ConA-induced acute liver injury was examined (Figure 6A). As shown in Figure 6B, transferred *Ccr9^–/–^* pDCs migrated to the inflamed liver more efficiently than the WT pDCs, despite transfer of an equal number of cells. Consequently, the adoptive transfer of *Ccr9^–/–^* pDCs was found to exert an enhanced immunosuppressive effect compared with that exerted by WT pDCs, as revealed by histological and serological analyses (Figure 6, C–E). Furthermore, consistent with these findings, serum IFN-γ levels were also significantly decreased by the transfer of *Ccr9^–/–^* pDCs (Figure 6F). Notably, supplementation of *Ccr9^–/–^* pDCs also protected the mice from acute liver injury even up to 8 hours following ConA administration (Figure 6, G–I). These results provide further evidence in favor of improved pDC migratory potential to the liver and subsequent protection against acute liver injury due to the manipulation of the CCR9/CCL25 axis.

## Discussion

ALF is a life-threatening condition characterized by progressive and extensive multilobular damage to hepatocytes accompanied by massive intrahepatic infiltration of immune cells. However, no definitive therapeutic options for ALF apart from liver transplantation are currently available. The increasing demand for liver transplantation and insufficient availability of donors highlight the requirement of alternative therapeutic options for this condition. The present study demonstrates the potential of manipulating the CCR9 axis as a novel approach to increase the efficiency of immunosuppressive pDC migration to the inflamed liver and the subsequent therapeutic benefits in acute liver injury. These findings corroborate previous reports on the potential utilization of immunosuppressive pDCs as a therapeutic strategy against ALF.

A greater abundance of pDCs was observed in the PB and liver of *Ccr9^–/–^* mice than in those of WT mice under steady-state and inflammatory conditions. Additionally, the absence of CCR9 improved the migration of pDCs to the liver under steady-state conditions. Although chemokines are responsible for the migration of pDCs to specific organs, as exemplified by the roles played by CXCR4, CXCR3/CCR5/CCR7, CCR7/CXCR4, and CCR9 in their migration to the BM, lymph nodes, spleen, and gut, respectively (27, 29–31), those involved in their migration to the liver are unknown. *Ccr9^–/–^* mice exhibited significantly decreased pDC homing to the small intestine, and a compensatory increase in peripheral circulation and liver accumulation, suggesting that the migration of pDCs to the liver may be independent of the CCR9/CCL25 axis. The expression of CCL25, which is generally higher in the small intestine, might regulate the dependency of the migration of pDCs in each organ.

Furthermore, we demonstrated that CCR9 deficiency does not affect the immunosuppressive function of pDCs, as indicated by the unchanged expression profiles of immunosuppressive genes as well as suppression of T cell proliferation in vitro. Notably, the transfer of *Ccr9^–/–^* pDCs into mice with ConA-induced liver injury resulted in efficient migration of pDCs to the liver and enhanced protection against hepatic inflammation. Although CCR9 is essential for pDC maturation and its downregulation has been reported upon pDC activation (10), our findings suggest that CCR9 expression does not determine the immunosuppressive ability of pDCs but rather affects their distribution within the body on the basis of inflammation. Hence, the protection against acute liver injury by *Ccr9^–/–^* pDCs was a consequence of increased migration of pDCs to the liver and not on account of functional modifications.

Recently, we reported that inhibition of the CCR9/CCL25 axis ameliorates acute hepatitis and liver fibrosis via regulation of hepatic macrophages (28, 32–34). The present study supports the notion that the combined use of a CCR9 inhibitor and pDC-based treatment may result in a better therapeutic outcome owing to their synergistic effects. The suppression of acute liver injury observed in *Ccr9^–/–^* mice may, therefore, be partly due to the increased number of immunosuppressive pDCs and the suppressed number/function of inflammatory macrophages.

Although our findings have potential clinical application, not much is known about how the CCR9/CCL25 axis affects the migration/distribution of pDCs in humans. In the context of CCR9-dependent pDC distribution in mice, we successfully confirmed the upregulation of CCL25 in human small intestines from patients with Crohn’s disease (Supplemental Figure 4), which is consistent with a previous report on the abundance of gut-tropic pDCs in patients with inflammatory bowel disease (35). It may be important to know whether CCR9 inhibition or intestinal resection would result in the hepatic accumulation of pDCs in humans, especially for patients with primary sclerosing cholangitis, 70% of whom have complications of inflammatory bowel disease. Nonetheless, further investigations are essential to elucidate the regulatory factors involved in the migration of pDCs, particularly to the human liver. Additionally, it is crucial to recognize that the manipulation of chemokines or their receptors may potentially exacerbate pDC-induced pathologies, including psoriasis (36) and systemic lupus erythematosus (37).

In conclusion, the present study demonstrates that the inhibition of CCR9 in pDCs may augment their protective effect against acute liver injury by enhancing their migration to the diseased organ. Although further validation is essential, regulation of chemokines or receptors in the CCR9/CCL25 axis may potentially be employed as a pDC-based therapeutic strategy for acute liver injuries in the future.

## Methods

### Mice.

C57BL/6 mice were procured from CLEA Japan Inc. *Ccr9^–/–^* mice with C57BL/6 background used in the study have been previously described (38). *Siglech^dtr/dtr^* mice with C57BL/6 background had been established previously (39). C57BL/6-Ly5.1 mice were obtained from Taconic Biosciences. *Abcb4^–/–^* (MDR2^–/–^) mice in FVB.129P2 background were obtained from The Jackson Laboratory. The mice were maintained under specific pathogen–free conditions in the Animal Care Facility at Keio University School of Medicine.

### Isolation of mouse tissue–derived immune cells.

Liver mononuclear cells (MNCs) were isolated as previously described (40). Briefly, the livers were perfused with PBS via the portal vein, minced, and passed through a 100 μm nylon mesh. The filtrate was then centrifuged at 50*g* for 1 minute followed by washing of the supernatant once. BM cells were hemolyzed and passed through a 100 μm nylon mesh. The S-IE fraction was prepared by digestion of intestinal tissues with HBSS (Nacalai Tesque) containing 1 mM DTT (Sigma-Aldrich) and 5 mM EDTA (Gibco) for 30 minutes at 37°C. Liver, PB, BM, and S-IE cells were suspended in 40% Percoll and overlaid onto 75% Percoll. Gradient separation was performed by centrifugation at 840*g* for 20 minutes at 20°C. MNCs that settled at the interphase were washed and resuspended in FACS buffer or RPMI 1640 medium (Sigma-Aldrich) containing 10% FBS and 1% penicillin/streptomycin (Nacalai Tesque).

### Flow cytometry and cell sorting.

Cells were blocked with anti-FcR (CD16/32, BD Biosciences) for 5 minutes, followed by incubation with the specific fluorescence-labeled antibodies at 4°C for 20 minutes. The antibodies anti–mouse CD45 (BV421/BV510, clone 30-F11), anti–mouse CD45.2 (BV510, clone 104), anti–mouse CD45.1 (FITC, clone A20), anti–mouse CD11b (APC-Cy7, clone M1/70), and anti–mouse CD11c (FITC/PE-Cy7, clone HL3) were obtained from BD Biosciences; anti–mouse B220 (PerCP-Cy5.5, clone RA3-6B2), anti–mouse PDCA-1 (APC, clone 129c1), and anti–mouse Siglec-H (PE, clone 551) from BioLegend; and anti–mouse CCR9 (FITC/BV421, clone eBioCW1.2/CW1.2) from Thermo Fisher Scientific/BD Biosciences. Events were acquired with a FACSCanto II instrument (Becton Dickinson) and analyzed using FlowJo software (Tree Star Inc.). Cell sorting was performed using FACSAria (Becton Dickinson) along with confirmation of >95% purity of the sorted cells.

### RNA sequencing.

Total RNA was isolated from sorted pDCs derived from the liver, BM, and S-IE using TRIzol reagent (Thermo Fisher Scientific K.K.) and Direct-zol RNA MicroPrep (Zymo Research). RNA-Seq was performed using SMART-Seq II (41). In brief, total RNA samples were mixed with oligo-dT and deoxyribose nucleoside triphosphates. This was followed by incubation at 72°C before placement of the mixtures on ice. The samples were spun down and reverse-transcribed into cDNA using the polyA tail. The template was switched at the 5′ end of the RNA, and full-length cDNA was amplified using PCR. After tagmentation-based library construction, PCR products were purified and selected using the Agencourt AMPure XP-Medium kit. These were subsequently heat-denatured and circularized using the splint oligonucleotide sequence. Single-stranded circular DNA was then used as the final library. Libraries were sequenced using BGI DNBSeq in 100 bp paired-end mode. The sequenced reads were mapped to the mouse reference genome (NCBI mm10), and read counts were determined using the Salmon version 0.14.1 software pipeline (42). Normalization of read counts to the trimmed mean of M (TMM) values by edgeR, heatmap, and principal component analysis plot was determined using the TCC-GUI tool (https://github.com/swsoyee/TCC-GUI) and R package. Pearson’s correlation matrix was generated using the iDEP.91 tool (http://bioinformatics.sdstate.edu/idep/) for the top 75% of genes. Gene Ontology (GO) enrichment analysis was performed using the clusterProfiler tool (https://bioconductor.org/packages/release/bioc/html/clusterProfiler.html) and R package.

### Preparation of BM-derived DCs.

BM lymphocytes were obtained by hemolysis of BM cell suspensions followed by filtration through a 100 μm nylon mesh. The cells (1 × 10^6^ cells/mL/6-well dish) were then cultured in pDC-conditioned medium (RPMI 1640, Sigma-Aldrich) containing 10% FBS, 1% penicillin/streptomycin, 10 mM HEPES, 1× MEM Non-Essential Amino Acids, 55 μM 2-mercaptoethanol (Gibco), and 100 ng/mL mouse recombinant FMS-like tyrosine kinase 3 ligand (FLT3L; PeproTech or Cell Guidance Systems Ltd.). After 8 days of culture, nonadherent cells were harvested. For in vivo adoptive transfer and in vitro T cell suppression assays, B220^+^ cells were separated using magnetic beads (Miltenyi Biotec) per the manufacturer’s recommendations. Greater than 95% purity of CD45^+^CD11b^–^B220^+^PDCA-1^+^Siglec-H^+^ cells was confirmed by flow cytometry before subsequent experiments. For in vitro quantitative reverse transcription PCR assays, cells were further purified to >98% purity by cell sorting on a FACSAria instrument (Becton Dickinson) using B220 and Siglec-H staining.

### RT-qPCR.

Total RNA was extracted from cells using the Direct-zol RNA MicroPrep Kit (Zymo Research). cDNA was synthesized by reverse transcription using the iScript cDNA Synthesis Kit (Bio-Rad). For quantification, real-time PCR was performed using the StepOne Plus System (Thermo Fisher Scientific) with the TaqMan Universal Master Mix and the following predesigned probes: *Ccr9* (Mm02528165_s1), *Il10* (Mm01288386_m1), *Il12a* (Mm00434165_m1), Epstein-Barr virus–induced gene 3 (*Ebi3*; Mm00469294_m1), *Il27p28* (Mm00461164_m1), TGF-β1 (*Tgfb1*; Mm01178820_m1), *Ifna1/5/6* (Mm03030145_gH), and *Ifnb1* (Mm00439552_s1). Target gene expression was normalized to that of *GAPDH*.

### In vitro T cell suppression assay.

CD4^+^CD25^–^ Teffs were isolated from splenocytes using a CD4^+^CD25^+^ regulatory T cell isolation kit (Miltenyi Biotec) following the manufacturer’s recommendations. FLT3L-proliferated pDCs from WT and *Ccr9^–/–^* mice were isolated as described in the subsection *Preparation of BM-derived DCs*. To evaluate the effect of pDCs on Teff proliferation, they were stained with a diluted solution of Violet Proliferation Dye 450 (VPD450; BD Biosciences) at 37°C for 15 minutes. FLT3L-proliferated pDCs from WT or *Ccr9^–/–^* mice (1 × 10^5^, 0.5 × 10^5^, or 0.25 × 10^5^ cells per well) were then cocultured with the VPD450-stained Teffs (1 × 10^5^ cells per well) and stimulated with 2 μL/well of Dynabeads mouse T-activator CD3/CD28 for T cell expansion and activation (Thermo Fisher Scientific) at 37°C for 96 hours. After incubation, the cells were washed, and cell proliferation was assessed using flow cytometry. T cell suppression was estimated on the basis of the rate of nonproliferated Teffs, where stimulated Teffs were considered as 0% suppressed cells, and nonstimulated Teffs were considered as 100% suppressed, along with the value of CD3/CD28.

### ConA-induced hepatitis mouse model.

ConA type IV (Sigma-Aldrich) was injected i.v. into the tail vein of mice (6- to 8-week-old males) 18 hours before the study endpoint at a dose of 15 mg/kg.

### CCl_4_-induced hepatitis model.

CCl_4_ (FUJIFILM Wako Pure Chemical Corporation) in corn oil was injected intraperitoneally (6- to 8-week-old males) 20 hours before the study endpoint at a dose of 1 mL/kg.

### Measurement of liver injury.

Serum alanine aminotransferase (ALT), aspartate aminotransferase (AST), alkaline phosphatase, and total bilirubin levels were measured using a Fuji DRI-CHEM analyzer (Fujifilm), according to the manufacturer’s instructions. Livers were fixed in 10% formalin and embedded in paraffin. The sections were stained with H&E before examination.

### Measurement of IFN-γ concentration.

IFN-γ concentrations in the culture supernatant and serum were determined using the Cytometric Bead Array Mouse Inflammation Kit (BD Biosciences) according to the manufacturer’s recommendations.

### Immunohistochemistry of human tissues.

Three diseased ileal samples were obtained from surgically resected patients with Crohn’s disease, and 2 normal ileal samples were acquired from surgically resected colon cancer patients from 2019 to 2020. Written informed consent was obtained from all patients. The samples were fixed in 10% formalin and embedded in paraffin blocks. Sections of 4to 5 μm thickness were prepared and stained with H&E, followed by immunohistochemical staining for CCL25. Immunohistochemical staining with the anti-CCL25 antibody (catalog 500-P134, rabbit polyclonal, dilution 1:250; PeproTech) was performed using an automated Bond RXm stainer (Leica Biosystems). This was accomplished using the heat-induced antigen retrieval method in pH 9 EDTA solution at 100°C for 10 minutes as instructed by the manufacturer.

### Data availability.

RNA-Seq data were deposited in the DNA Data Bank of Japan and are publicly available at accession number DRA014583.

### Statistics.

Statistical analyses were performed using GraphPad Prism software (version 7.0, GraphPad Software Inc.). Differences between 2 groups were evaluated using the 2-sided unpaired Student’s *t* test. Comparisons of more than 2 groups were performed using 1-way ANOVA, followed by Tukey-Kramer multiple-comparison test. For all analyses, significance was accepted at the 95% confidence interval level (*P* < 0.05).

### Study approval.

All experiments involving animals were approved by the regional animal study committee (Animal Ethics Committee of Keio University, Tokyo, Japan) and performed according to institutional guidelines and home office regulations. The institutional review board of Keio University School of Medicine approved all human studies (no. 20170255) according to the guidelines of the 1975 Declaration of Helsinki (2008 revision). Written informed consent was obtained from all participants.

## Author contributions

YK conceived and designed the study, performed most experiments, analyzed the data, and wrote the manuscript. NN conceived of and drafted the manuscript. PSC, NT, and MS participated in scientific and technical discussions. TT, AU, TA, SC, and KM performed experiments. TK supervised the study.

## Supplementary Material

Supplemental data

## Version 1 08/09/2022

In-Press Preview

## Version 2 09/08/2022

Electronic publication
