# Supplementary material for: CCR9 axis inhibition enhances hepatic migration of plasmacytoid DCs and protects against liver injury
Source: JCI Insight. 2022 Sep 8;7(17):e159910. doi: 10.1172/jci.insight.159910 (PMC9536268; doi:10.1172/jci.insight.159910)
Supplement: Supplemental data [file jciinsight-7-159910-s114.pdf]

Supplementary Information for

CCR9 axis inhibition enhances hepatic migration of plasmacytoid dendritic cells and protects against liver injury

Yuzo Koda,[1,2] Nobuhiro Nakamoto,[1*] Po-Sung Chu,[1] Toshiaki Teratani,[1] Akihisa Ueno,[3] Takeru Amiya,[1,2] Nobuhito Taniki,[1] Sayako Chiba,[1] Kentaro Miyamoto,[1] Michiie Sakamoto,[3] Takanori Kanai[1,4*]

[1]Division of Gastroenterology and Hepatology, Department of Internal Medicine, Keio University School of Medicine, Tokyo, Japan
[2]Mitsubishi Tanabe Pharma Corporation, Kanagawa, Japan
[3]Department of Pathology, Keio University School of Medicine, Shinanomachi, Tokyo, Japan
[4]Japan Agency for Medical Research and Development, AMED, Tokyo, Japan

*Address correspondence to Nobuhiro Nakamoto (nobuhiro@z2.keio.jp, +81-3353-1211) and Takanori Kanai (takagast@z2.keio.jp, +81-3353-1211), Division of Gastroenterology and Hepatology, Department of Internal Medicine, Keio University School of Medicine, Tokyo 1608582, Japan.

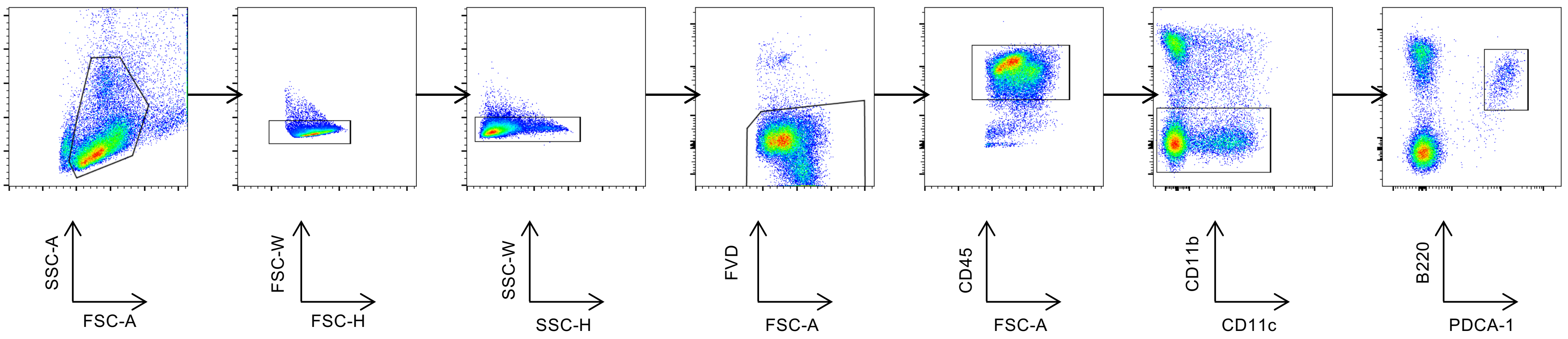

Supplemental Figure 1. The gating strategy of FACS analysis.
Isolated mononuclear cells from various organs were analyzed.

Figure S1 Koda et. al.

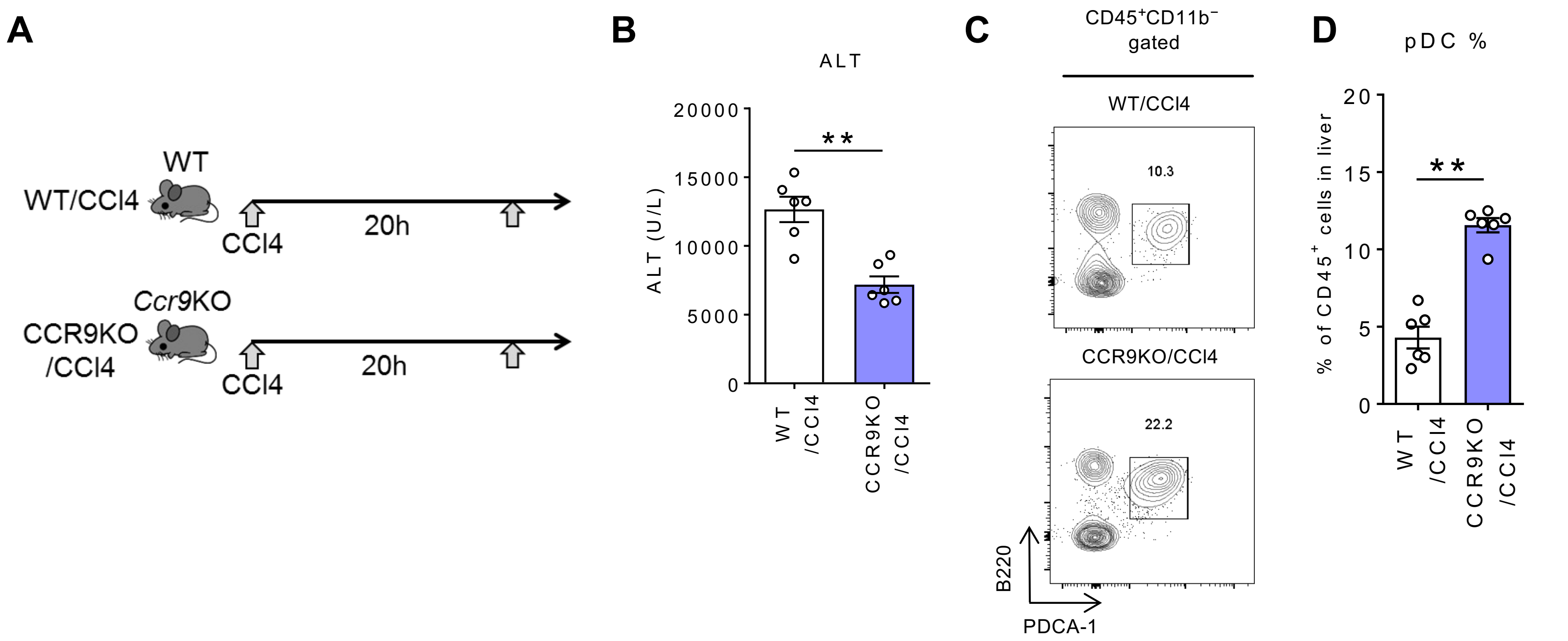

Supplemental Figure 2. CCR9 deficiency induces accumulation of pDCs in the liver under CCl4-induced inflammation. (A) Study design. WT or Ccr9-/- mice were intraperitoneally injected with CCl4 (1 ml/kg) in corn oil or corn oil. All mice were sacrificed and analyzed 20 h following Ccl4 injection. (B) Serum ALT levels. (C) Representative B220 and PDCA-1 staining of CD45+CD11b--gated liver MNCs. (D) Mean percentages of pDCs in the liver during CCl4-induced hepatitis. Data represent the mean ± SEM (n = 6 per group). **p < 0.01, Student's t-test. Data are combined from two independent experiments.

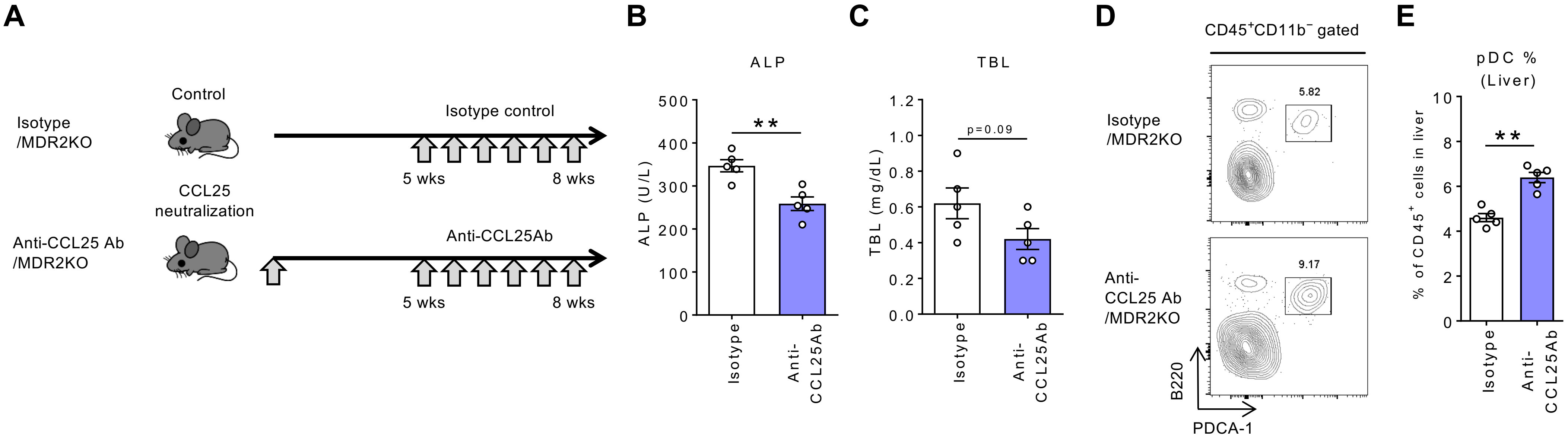

Supplemental Figure 3. CCL25 neutralization induces accumulation of pDCs in the liver under MDR2 deficiency-induced cholestasis.
(A) Study design. Mdr2-/- mice (FVB background) were intravenously injected with anti-CCL25 antibody (100 µg/mouse, R&D, clone 89818) or isotype control (100 µg/mouse, BioXCell, clone LTF-2) in PBS twice per week. All mice were sacrificed and analyzed 3 weeks after the first injection of the antibody at 5 weeks of age. (B) Serum ALP levels. (C) Serum total bilirubin levels. (D) Representative B220 and PDCA-1 staining of CD45+CD11b-gated liver MNCs. (E) Mean percentages of pDCs in the liver. Data represent the mean ± SEM (n = 5 per group). **$p < 0.01$, Student's t-test. Data are combined from two independent experiments.

Figure S3 Koda et. al.

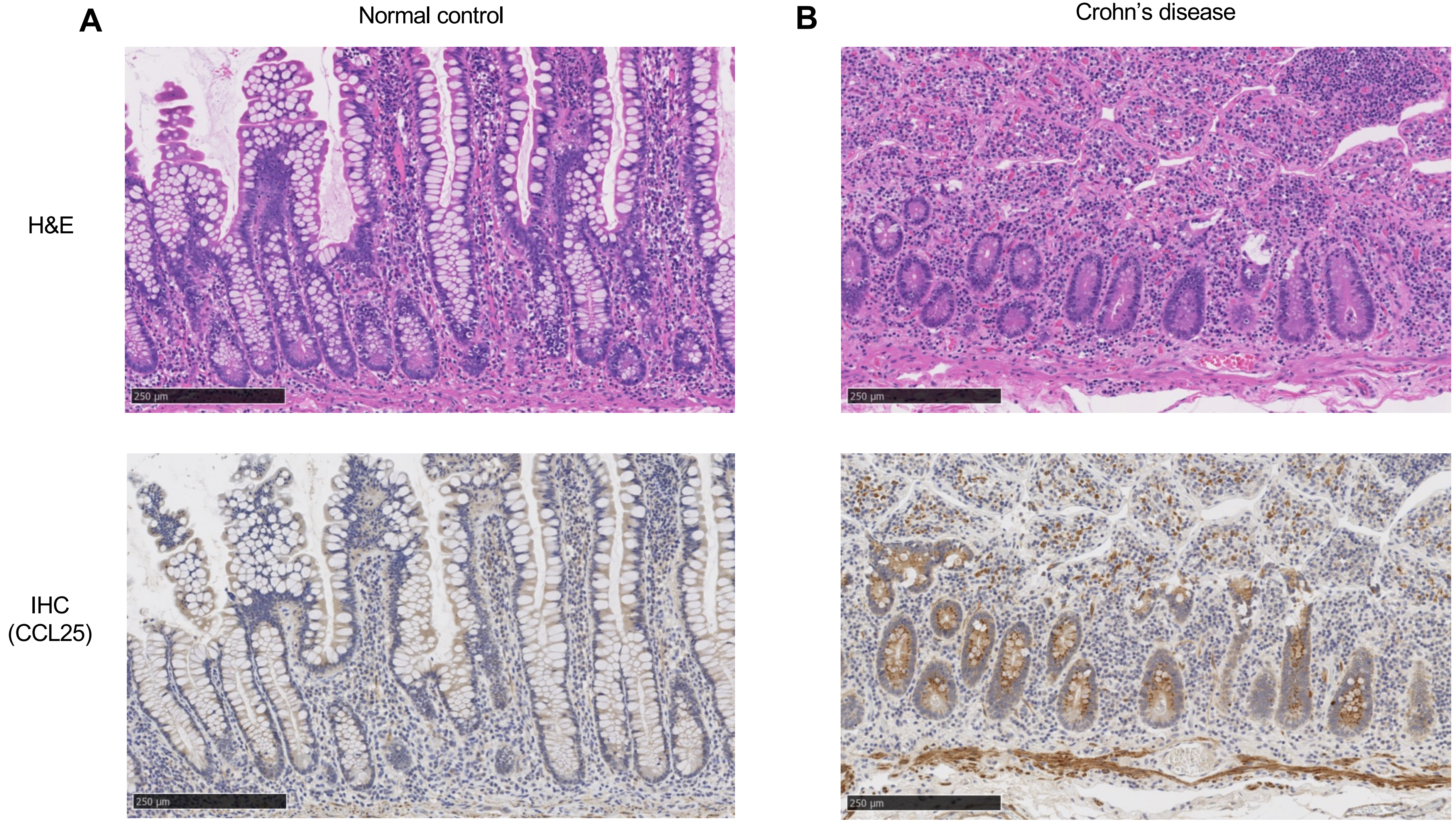

Supplemental Figure 4. CCL25 is expressed in the small intestinal epithelium of healthy controls and patients with Crohn's disease.

Representative photomicrographs of H&E-stained (upper) and anti-CCL25 Ab–stained (lower) IHC sections of the small intestine. (A) The normal control derived from the colon cancer patient. (B) The patient with Crohn's disease. Scale bars: 250 µm.
